# Investigating possible shared single nucleotide polymorphisms in isolated oral cleft and non-cleft facial morphology

**DOI:** 10.3389/fdmed.2025.1546295

**Published:** 2025-04-15

**Authors:** Erika Calvano Küchler, Michelle Nascimento Meger, Bruna Correia Rauta Pires, Svenja Beisel-Memmert, Daniel Hemming, Ricardo D. Coletta, Rafaela Scariot, Mírian Aiko Nakane Matsumoto, Maria Angelica Hueb de Menezes Oliveira, Christian Kirschneck, Bianca Cavalcante-Leão

**Affiliations:** ^1^ Department of Orthodontics, Medical Faculty, University Hospital Bonn, Bonn, Germany; ^2^ School of Dentistry, Tuiuti University of Paraná, Curitiba, Paraná, Brazil; ^3^ Department of Oral Diagnosis and Graduate Program in Oral Biology, School of Dentistry, University of Campinas, Piracicaba, São Paulo, Brazil; ^4^ Department of Stomatology, Federal University of Paraná, Curitiba, Paraná, Brazil; ^5^ Department of Pediatric Dentistry, School of Dentistry of Ribeirão Preto, USP—São Paulo University, Ribeirão Preto, São Paulo, Brazil; ^6^ Department of Biomaterials, University of Uberaba, Uberaba, Minas Gerais, Brazil

**Keywords:** face, genes, polymorphism, cleft lip and/or palate, craniofacial development

## Abstract

**Introduction:**

Facial morphogenesis is regulated by several cellular interactions that are mediated by numerous morphogenetic signals. Based on the existing evidence, we hypothesize that oral cleft-associated single nucleotide polymorphisms (SNPs) are involved in the normal range of human face development. Therefore, this study aimed to investigate the association between SNPs in oral cleft-related genes and variations in the normal range of facial morphology.

**Method:**

A sample of healthy Brazilian teenagers (aged between 11 and 18 years old) were screened and collected. Frontal facial digitized photographs from orthodontic records were used to determine phenotypes, while the DNA extracted from saliva samples was used to investigate the candidate SNPs. Five oral cleft-associated SNPs in *BMP2* (*rs235768*), *BMP4* (*rs17563*), *WNT3A* (*rs708111*), *WNT11* (*rs1533767*), and *RUNX2* (*rs1200425*) were selected, and allelic discrimination analysis was performed using real-time PCR.

**Results:**

A total of 58 individuals (27 boys and 31 girls) were included. The facial landmarks used for the facial measurements were the trichion (Tr), glabella (G), nassion (N), subnasale (Sn), labrale superior (Ls), labrale inferior (Li), gnathion (Gn), cheilon (Ch), and zygoma (Zg). *rs17563* in *BMP4* was associated with lip proportion, in which individuals with the homozygous GG genotype had a higher Ch-Ch:Ls-Li proportion than the heterozygous AG genotype (*p* = 0.034). rs1533767 in *WNT11* was associated with G-Sn:Sn-Gn (*p* = 0.028), N-Gn:Sn-Gn (*p* = 0.035), and Sn-Gn:Tr-Gn (*p* = 0.039).

**Conclusion:**

Our study supported the hypothesis that oral cleft-associated SNPs are involved in the normal range of human facial morphology.

## 1 Introduction

The human face displays high morphological variability. Its appearance is a fascinating trait, and facial variability is important for social interactions. Facial variability also has implications for clinical practice, forensic intelligence identification, personal identity, clinical genetics, and possible counseling. Human faces result from the intrinsic complexity of morphogenesis, in which many genetic and environmental factors are involved. Genetics is clearly one of these factors, and this knowledge is long supported by the evidence on familial similarities, especially for monozygotic twins (1).

Some studies have emerged in the past two decades that aimed to unravel the genes that may play a critical role in defining the size and shape of facial features (2–7). Certain studies explored the connection between oral cleft-related genes and normal-range variations in facial morphology (1–8). These studies have provided some important insights into genes and single nucleotide polymorphisms (SNPs) in facial genetics (1). SNPs are a type of polymorphism involved in a variation of a single base pair at a single position in a DNA sequence. SNPs located in several protein encoding genes have already been associated with non-syndromic (isolated) oral cleft (9–14).

Facial morphogenesis is regulated by several cellular interactions that are mediated by numerous morphogenetic signals, such as bone morphogenetic protein (*BMP*), wingless (*WNT*) (15), and Runt-related transcription factor 2 (*RUNX2*) (16). *BMPs* are well-known as multi-functional growth factors. *BMP* signaling is one of the most important pathways regulating craniofacial development. It is involved in the early development of the head and facial patterning (17), in which their components are important factors in the growth of facial processes. Animal models showed that *BMP* signaling has distinct roles in lip and palate fusion (18), especially *BMP2* and *BMP4,* which are expressed in maxillary and mandibular processes (19). SNPs in *BMP2* (11, 12) and *BMP4* (10, 11, 13) have been associated with oral clefts in different populations.

Canonical *WNT* signaling factors play a decisive role in many aspects of craniofacial development, while their dysregulation is involved in facial congenital defects (20). In animal models, *WNT3A* was involved in malformations of the face (2, 20) due to increased cell proliferation in the mesenchyme and increased expression of *BMP2* and *BMP4* (21). *WNT11* is also important for craniofacial development, especially the palate area (22). In fact, SNPs in *WNT3A* and *WNT11* have been associated with oral cleft in humans (9). *RUNX2* is a critical regulator of transcription processes and involved in craniofacial development, in bone and dental formation in particular, and was furthermore connected to cleft palates in mice (23). A study that examined the association between 49 genetic variants in *RUNX2* and oral cleft in different populations observed that some SNPs in this gene are associated with oral cleft (24).

The identification of SNPs associated with normal facial morphology is a valuable tool for advancing medical, scientific, and applied fields. This knowledge improves the understanding of the biological processes underlying facial structure, growth, and variation. Understanding the genetic basis of facial morphology may lead to personalized surgical or orthodontic interventions, ensuring better functional and esthetic outcomes. SNP data associated with facial features could enhance forensic reconstruction, allowing for more accurate predictions of an individual's appearance from DNA samples. SNPs linked to facial morphology can offer insights into human evolution, population genetics, and how environmental and genetic factors have shaped human facial diversity. In addition, understanding the role of oral cleft-associated SNPs in normal facial traits is crucial for identifying parents who may be at a higher risk of having a child with an oral cleft. Therefore, in the present study, we selected SNPs previously associated with oral cleft (rs235768, rs17563, rs708111, rs1533767, and rs1200425) to investigate their role in normal facial morphology. Our hypothesis is that oral cleft-associated SNPs are involved in the normal range of human face development.

## 2 Methods

### 2.1 Ethical aspects and sample description

The ethical rules for research described in the Helsinki Declaration (1964) were followed throughout the study. All included individuals provided written informed consent, and the study protocol was approved by the Ethics Committee of the School of Dentistry of Ribeirão Preto, University of São Paulo, São Paulo, Brazil (Protocol No. 50765715.3.0000.5419). Children who had parental consent were then asked for assent before they were included in the study. Only children with both informed consent and assent were included in the screening.

This study is part of a larger project regarding the etiological aspects involved in craniofacial development. For this study, frontal facial digitized photographs from the orthodontic records were used to determine the phenotypes, while the DNA samples were used to investigate the candidate SNPs. During the recruiting process, only biologically unrelated healthy teenagers or young adults who sought orthodontic treatment at the School of Dentistry of Ribeirão Preto, University of São Paulo, were included. The exclusion criteria were based on the clinical examination performed by dentists and the self-reported information acquired during the anamnesis. Patients were excluded if they had syndromes, had received or were receiving hormonal treatment, presented with congenital alterations such as cleft lip and/or palate or oligodontia (congenital absence of six or more teeth), had first-degree relatives with cleft lip and/or palate, had undergone previous orthodontic and/or orthopedic treatments, had a history of or required orthognathic surgery, or had facial trauma or endocrinological problems such as growth hormone deficiency, thyroid disorders, and diabetes mellitus. During clinical examination, none of the patients had permanent teeth extracted.

The sample size calculation was based on the frequency of the most common allele, an expected mean difference of 0.3 among genotypes, and a standard deviation of 0.3, with an established alpha level of 5% and a power of 80%.

### 2.2 Phenotyping

The photographs were taken with the patient positioned under standardized conditions in portrait mode (non-smiling). Briefly, the patient was standing upright with a natural posture. Their hair was pulled back to ensure full visibility of facial features, and glasses, earrings, and any accessories that could obstruct facial landmarks were removed. The camera was positioned at the patient's eye level. The patient faced the camera directly with their head in a natural head position and the Frankfort Horizontal Plane parallel to the floor. The patient should have a relaxed facial expression in a natural rest position for the photograph. All photographs were taken in high resolution with the correct exposure settings.

All frontal facial digitized photographs were available in the orthodontic record and only the images from before the orthodontic treatment were used.

**Figure 1:**
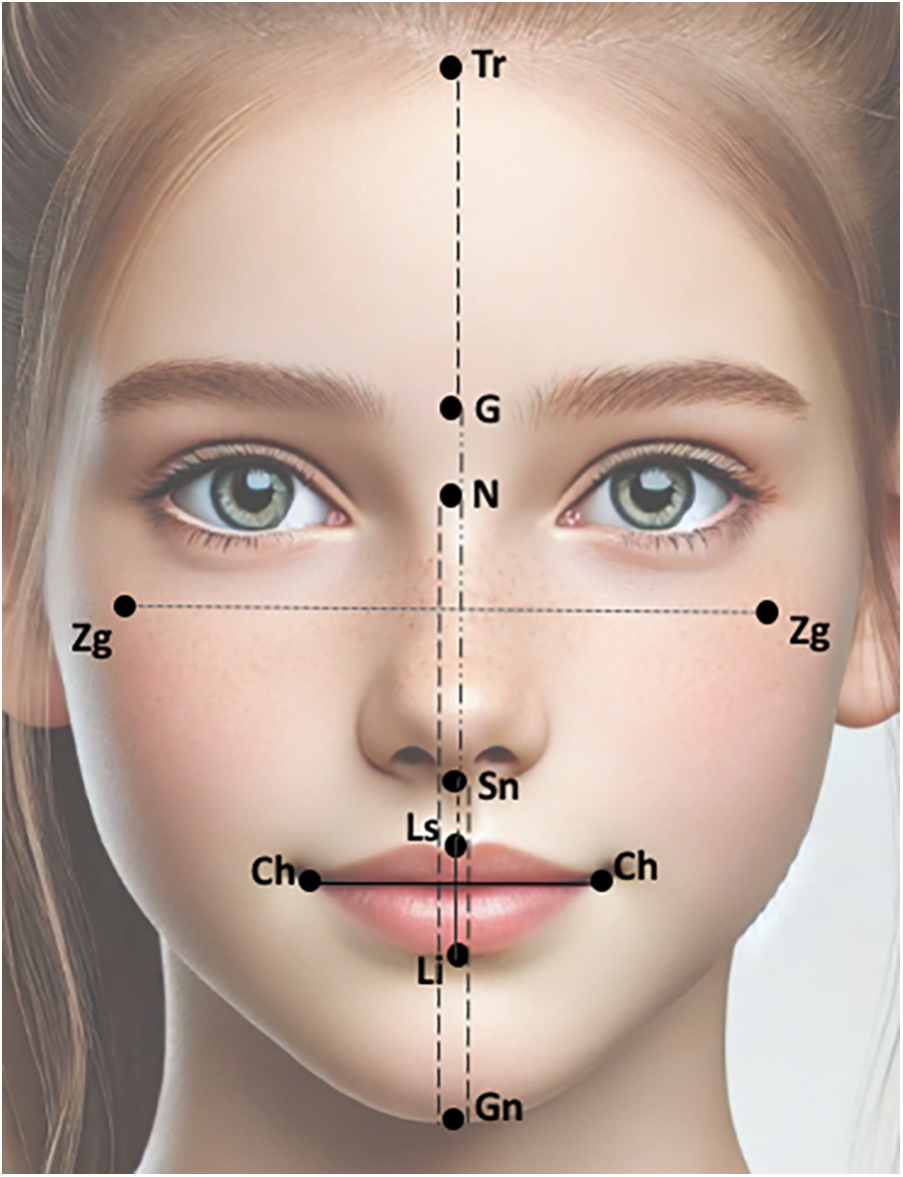
Facial landmarks and linear measurements used in the present study. The figure was adapted from an AI-generated image created using ChatGPT (January 2025, version 4), OpenAI.

A total of 15 facial landmarks (7 single and 4 paired) were selected for this study. The landmarks were selected based on their reliable identification in all photographs, and being minimally affected by changes due to the patients’ grooming (i.e., eyebrow plucking or hair styling). The facial landmarks used for the facial measurements were the trichion (Tr), glabella (G), nassion (N), subnasale (Sn), labrale superior (Ls), labrale inferior (Li), gnathion (Gn), cheilon (Ch), and zygoma (Zg). The description of the selected landmarks is presented in Table 1 and their locations are depicted in Figure 1. The position of each landmark was recorded as a set of X-Y pixel coordinates and to place the landmarks on the 2D images, Adobe Photoshop Creative Cloud (Adobe Systems Incorporated, San Jose, CA, USA) was used. The edited photographs were imported into the Image J software (NIH, Bethesda, MD, USA) and the distance between any two points in a given plane of space was accomplished by calculating the number of pixels that existed between the two coordinates. The measurements were Tr-G, G-Sn, Sn-Gn, N-Gn, Zg-Zg, Ls-Li, Ch-Ch, Sn-Ls, and Li-Gn. The ratios were calculated for each individual.

**Table 1:** Facial landmarks used.

Abbreviation	Landmark
Tr	Trichion
G	Glabella
N	Nassion
Sn	Subnasale
Ls	Labrale superior
Li	Labrale inferior
Gn	Gnathion
Ch	Cheilion
Zg	Zigoma

### 2.3 Reliability

To ensure the accuracy and reproducibility of the measurements, both intra- and inter-observer reliability assessments were performed. The intra-class correlation coefficient (ICC) was used to evaluate the reproducibility and compared with that of the senior examiner. For the inter-observer reliability, the senior examiner and the trained examiner analyzed the same set of five randomly selected images under identical conditions. The ICC was used to determine the level of agreement between observers. There were no discrepancies to be resolved and the value for the inter-observer agreement was 0.87. For the intra-observer reliability, a single examiner conducted facial measurements twice on the same set of five randomly selected images (images different from the ones used in the inter-observer reliability stage), with a 2-week interval between assessments to minimize recall bias. The ICC was used to evaluate the consistency of repeated measurements and the value for the intra-observer agreement was 0.92. ICC values were interpreted as follows: <0.50 (poor), 0.50–0.75 (moderate), 0.75–0.90 (good), and >0.90 (excellent) reliability.

### 2.4 DNA extraction, genotyping, and quality control

DNA was extracted from buccal cells in saliva. For DNA extraction, an established previously published protocol was used (25) and the amount and purity of the DNA were assessed using spectrophotometry (Nanodrop 1000; Thermo Scientific, Wilmington, DE, USA). The DNA was stored until the polymerase chain reaction (PCR) analysis.

The oral cleft-associated SNPs in the candidate genes were selected from published association studies on isolated oral clefts, including a systematic review and meta-analysis. We only selected SNPs that are common in the global population (minor allele frequency > 0.25). A detailed description of the selected SNPs is provided in Table 2.

**Table 2:** Characteristics of the candidate SNPs.

Gene	SNP	Base change	Description of the studied SNPs
*BMP2*	rs235768	A/T	This SNP was associated with isolated oral cleft in two previous published studies (11, 12).
*BMP4*	rs17563	A/G	A systematic review showed that this SNP might be a risk factor for isolated oral cleft in the Chinese population and in the Brazilian population (13).
*WNT3A*	rs708111	A/G	Multiple haplotypes in Wnt genes, including haplotypes that include the SNP rs708111, were previously associated with isolated oral cleft (9).
*WNT11*	rs1533767	A/G	Multiple haplotypes in Wnt genes, including haplotypes that include the SNP rs1533767, were previously associated with isolated oral cleft (9).
*RUNX2*	rs1200425	A/G	This SNP was statistically significantly associated with isolated oral cleft. A study observed an under-transmission of the minor allele, which was associated with a decreased risk of oral cleft (24).

Allelic discrimination analysis was blindly performed using the Taqman™ method for real-time PCR (Step One Plus Real-Time PCR System, Applied Biosystems, Foster City, CA, USA).

Duplicated genotyping of 10% of the total sample was performed and 100% agreement was observed.

### 2.5 Statistical analysis

Deviations on the Hardy–Weinberg equilibrium (HWE) for each SNP were tested using the Chi-squared test.

The dependent variables were the ratio measurements, and the independent variables were the genotypes. The dimensions presented normal distribution according to the Kolmogorov–Smirnov test. The ratios were compared among the genotypes with a *t*-test or one-way ANOVA with Tukey’s *post-hoc* test. Data were analyzed using the GraphPad Prism Version 9.3.1. The established alpha for all analyses was 5%.

## 3 Results

A total of 58 individuals (27 boys and 31 girls) were included in this study after the screening and exclusion criteria process.

The age of the included individuals ranged from 11 to 18 years old, and the mean age was 13.5 years old (standard deviation = 1.83).

According to the genotypic data presented, we calculated the HWE in each SNP. Chi-square analysis was used to confirm that the genotype distribution of each evaluated SNP’s HWE. The results are as follows: chi-square ^HWE^ = 10.81 for rs235768, chi-square ^HWE^ = 2.63 for rs17563, chi-square ^HWE^ = 2.25 for rs708111, chi-square ^HWE^ = 0.10 for rs1533767, and chi-square ^HWE^ = 1.01 for rs1200425.

Table 3 shows the *p*-values and facial ratio proportion values (mean, standard deviation, minimum, and maximum) distributions according to the genotypes for each studied SNP (rs235768, rs17563, rs708111, rs1533767, and rs1200425).

**Table 3:** Dimension distributions according to the genotypes in the candidate SNPs.

Measures	Min–max	Mean (SD)	Min–max	Mean (SD)	Min–max	Mean (SD)	*p*-value
rs235768	AA (*n* = 0)		AT (*n* = 34)		TT (*n* = 19)		
Ch-Ch:Ls-Li	—	—	1.79–3.87	2.65 (0.44)	1.70–3.79	2.70 (0.60)	0.778
G-Sn:Sn-Gn	—	—	0.59–1.11	0.86 (0.10)	0.69–0.97	0.84 (0.08)	0.696
G-Sn:Tr-Gn	—	—	0.24–0.34	0.30 (0.01)	0.26–0.33	0.30 (0.01)	0.982
N-Gn:Sn-Gn	—	—	1.58–1.93	1.73 (0.09)	1.64–1.83	1.72 (0.06)	0.685
Sn-Gn:Li-Gn	—	—	1.09–3.50	2.02 (0.43)	1.56–2.77	2.14 (0.32)	0.294
Sn-Gn:Tr-Gn	—	—	0.30–0.40	0.35 (0.02)	0.32–0.40	0.35 (0.02)	0.645
Sn-Gn:Sn-Ls	—	—	2.30–6.86	3.75 (0.87)	2.56–5.26	3.84 (0.71)	0.713
Tr-G:G-Sn	—	—	0.89–1.41	1.13 (0.10)	0.93–1.36	1.12 (0.12)	0.771
Tr-G:Tr-Gg	—	—	0.28–0.37	0.34 (0.01)	0.29–0.37	0.33 (0.02)	0.696
Tr-Gn:Zg-Zg	—	—	1.31–1.60	1.45 (0.08)	1.25–1.62	1.45 (0.08)	0.889
rs17563	AA (*n* = 24)		AG (*n* = 18)		GG (*n* = 9)		
Ch-Ch:Ls-Li	1.79–3.79	2.71 (0.49)	1.70–3.12	2.47 (0.36)	2.27–3.87	2.96 (0.53)	**0**.**034**
G-Sn:Sn-Gn	0.69–1.11	0.87 (0.10)	0.59–1.08	0.83 (0.10)	0.78–0.91	0.85 (0.04)	0.499
G-Sn:Tr-Gn	0.26–0.34	0.30 (0.01)	0.24–0.33	0.29 (0.01)	0.27–0.32	0.30 (0.01)	0.593
N-Gn:Sn-Gn	1.60–1.91	1.74 (0.08)	1.58–1.93	1.71 (0.08)	1.60–1.79	1.71 (0.05)	0.478
Sn-Gn:Li-Gn	1.09–3.50	2.09 (0.52)	1.61–2.63	2.00 (0.24)	1.64–2.43	2.11 (0.24)	0.712
Sn-Gn:Tr-Gn	0.30–0.39	0.35 (0.02)	0.31–0.40	0.36 (0.02)	0.33–0.38	0.35 (0.01)	0.521
Sn-Gn:Sn-Ls	2.30–6.86	3.83 (1.09)	2.56–4.42	3.63 (0.47)	3.02–4.45	3.86 (0.51)	0.693
Tr-G:G-Sn	0.93–1.30	1.12 (0.10)	0.98–1.41	1.14 (0.10)	0.89–1.36	1.14 (0.13)	0.866
Tr-G:Tr-Gg	0.29–0.37	0.34 (0.01)	0.31–0.37	0.34 (0.01)	0.28–0.37	0.34 (0.02)	0.850
Tr-Gn:Zg-Zg	1.25–1.59	1.43 (0.08)	1.31–1.55	1.48 (0.08)	1.31–1.55	1.43 (0.09)	0.125
rs708111	AA (*n* = 15)		AG (*n* = 21)		GG (*n* = 17)		
Ch-Ch:Ls-Li	1.70–3.87	2.82 (0.60)	1.79–3.28	2.52 (0.35)	1.91–3.79	2.59 (0.48)	0.168
G-Sn:Sn-Gn	0.59–1.08	0.86 (0.11)	0.71–1.01	0.84 (0.07)	0.72–1.11	0.87 (0.10)	0.523
G-Sn:Tr-Gn	0.24–0.33	0.30 (0.02)	0.27–0.33	0.30 (0.01)	0.28–0.34	0.30 (0.01)	0.548
N-Gn:Sn-Gn	1.58–1.93	1.74 (0.10)	1.60–1.91	1.71 (0.07)	1.62 −1.90	1.74 (0.08)	0.438
Sn-Gn:Li-Gn	1.61–2.63	2.06 (0.31)	1.30–3.50	2.16 (0.44)	1.09–2.77	1.98 (0.38)	0.362
Sn-Gn:Tr-Gn	0.30–0.40	0.35 (0.02)	0.31–0.39	0.35 (0.02)	0.31–0.39	0.35 (0.02)	0.474
Sn-Gn:Sn-Ls	2.56–4.45	3.75 (0.52)	2.57–6.86	4.01 (0.97)	2.30–5.26	3.58 (0.77)	0.261
Tr-G:G-Sn	0.89–1.41	1.16 (0.13)	0.93–1.29	1.12 (0.08)	0.97–1.30	1.10 (0.09)	0.301
Tr-G:Tr-Gg	0.28–0.37	0.34 (0.02)	0.31–0.37	0.33 (0.01)	0.29–0.37	0.34 (0.01)	0.300
Tr-Gn:Zg-Zg	1.31–1.60	1.47 (0.09)	1.25–1.62	1.47 (0.08)	1.31–1.52	1.42 (0.07)	0.126
rs1533767	AA (*n* = 2)		AG (*n* = 15)		GG (*n* = 21)		
Ch-Ch:Ls-Li	2.31–3.00	2.55 (0.62)	1.70–3.87	2.79 (0.60)	2.07–3.57	2.62 (0.37)	0.559
G-Sn:Sn-Gn	0.86–0.95	0.90 (0.05)	0.70–1.11	0.90 (0.10)	0.59–0.95	0.81 (0.08)	**0**.**028**
G-Sn:Tr-Gn	0.29–0.32	0.31 (0.02)	0.27–0.34	0.30 (0.01)	0.24–0.31	0.29 (0.01)	0.096
N-Gn:Sn-Gn	1.72–1.89	1.81 (0.11)	1.59–1.91	1.76 (0.10)	1.58–1.81	1.70 (0.06)	**0**.**035**
Sn-Gn:Li-Gn	1.93–2.01	1.97 (0.05)	1.09–2.79	1.99 (0.43)	1.30–3.50	2.12 (0.44)	0.613
Sn-Gn:Tr-Gn	0.34–0.34	0.34 (0.00)	0.30–0.39	0.34 (0.02)	0.31–0.40	0.36 (0.02)	**0**.**039**
Sn-Gn:Sn-Ls	3.63–3.68	3.65 (0.03)	2.30–5.44	3.76 (0.82)	2.57–6.86	3.82 (0.86)	0.952
Tr-G:G-Sn	0.99–1.22	1.10 (0.16)	0.97–1.30	1.13 (0.10)	1.03–1.41	1.15 (0.09)	0.803
Tr-G:Tr-Gg	0.32–0.36	0.34 (0.02)	0.29–0.37	0.34 (0.02)	0.31–0.37	0.33 (0.01)	0.348
Tr-Gn:Zg-Zg	1.42–1.54	1.48 (0.08)	1.31–1.58	1.43 (0.09)	1.31–1.62	1.46 (0.09)	0.711
rs1200425	AA (*n* = 11)		AG (*n* = 21)		GG (*n* = 18)		
Ch-Ch:Ls-Li	2.00–3.87	2.72 (0.61)	1.70–3.57	2.70 (0.46)	1.91–3.79	2.61 (0.46)	0.815
G-Sn:Sn-Gn	0.72–0.97	0.83 (0.08)	0.59–1.11	0.85 (0.11)	0.69–1.08	0.87 (0.09)	0.606
G-Sn:Tr-Gn	0.27–0.32	0.29 (0.01)	0.24–0.34	0.30 (0.02)	0.26–0.33	0.30 (0.01)	0.699
N-Gn:Sn-Gn	1.62–1.88	1.72 (0.07)	1.58–1.90	1.71 (0.09)	1.64–1.93	1.74 (0.08)	0.536
Sn-Gn:Li-Gn	1.61–2.58	2.17 (0.31)	1.09–3.50	2.03 (0.52)	1.56–2.79	2.03 (0.26)	0.598
Sn-Gn:Tr-Gn	0.32–0.39	0.35 (0.02)	0.30–0.40	0.35 (0.02)	0.31–0.38	0.35 (0.02)	0.587
Sn-Gn:Sn-Ls	2.56–5.15	3.84 (0.73)	2.30–6.86	3.80 (1.02)	2.68–5.44	3.71 (0.63)	0.900
Tr-G:G-Sn	1.00–1.36	1.15 (0.10)	0.89–1.41	1.12 (0.12)	0.98–1.30	1.13 (0.09)	0.797
Tr-G:Tr-Gg	0.31–0.37	0.34 (0.01)	0.28–0.37	0.33 (0.02)	0.31–0.37	0.34 (0.01)	0.665
Tr-Gn:Zg-Zg	1.33–1.56	1.44 (0.07)	1.25–1.60	1.46 (0.10)	1.31–1.62	1.44 (0.08)	0.763

*T*-test was used for the rs235768 analysis. ANOVA with Tukey’s *post-hoc* test was used for the rs17563, rs708111, rs1533767 and rs1200425 analysis. Tr, trichion; G, glabella; N, nassion; Sn, subnasale; Ls, labrale superior; Li, labrale inferior; Gn, gnathion; Ch, cheilon; Zg, zygoma.

Bold values denote the difference between AG and GG.

**Figure 2:**
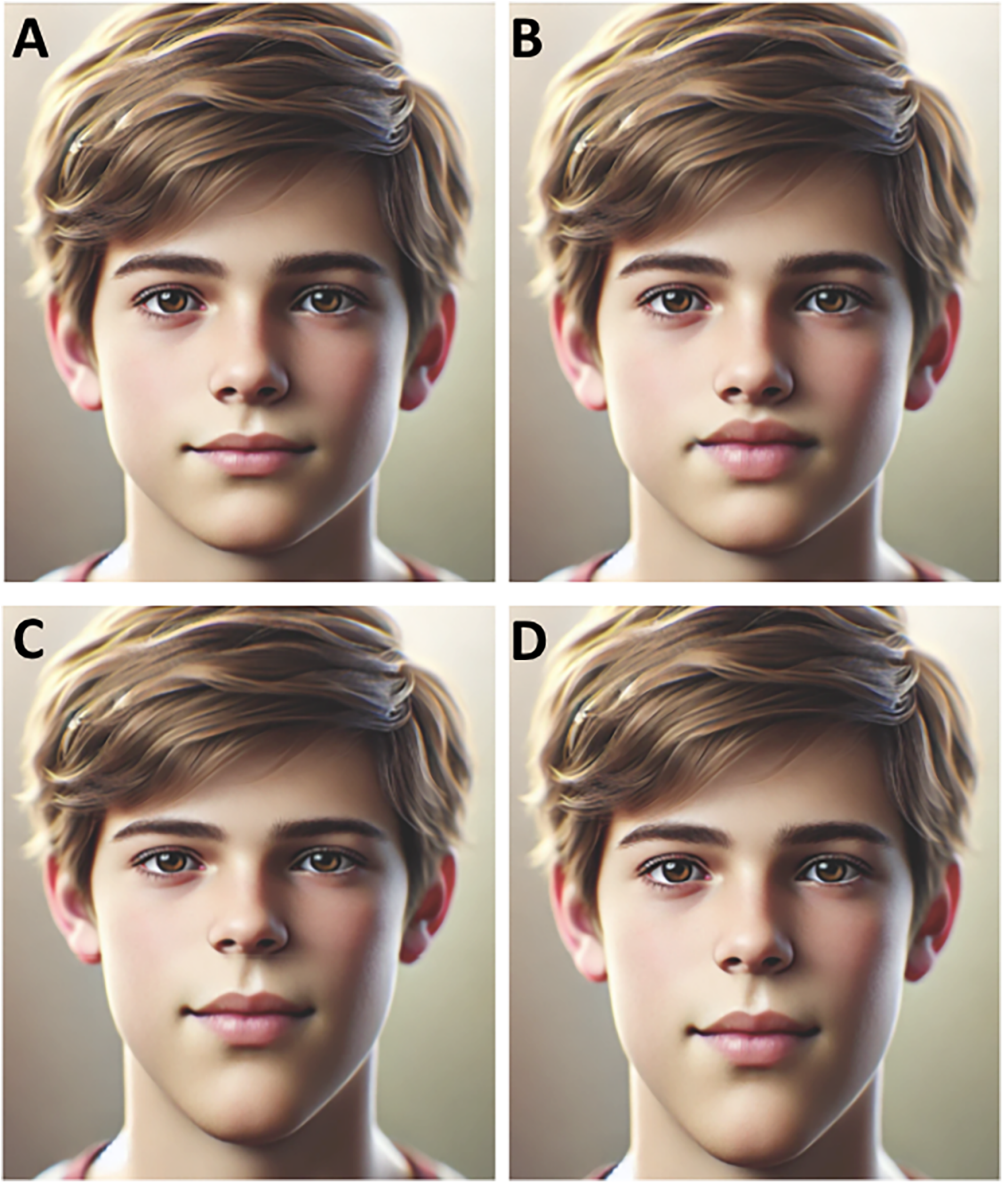
Schematics of different face morphologies associated with the genotypes in *BMP4* and *WNT1.* (**A**) A reference face with average proportions. (**B**) Face with thicker lips. (**C**) Face with a change in the mid- to lower facial height ratio, showing a larger lower face height. (**D**) Face with a changed ratio of lower face height to total face height, showing a larger lower face height. The figure was adapted from an AI-generated image created using ChatGPT (January 2025, version 4), OpenAI.

Figure 2 shows the differences in facial morphology with different facial proportions. The different facial proportions represent the influence of the genotypes in *BMP4* and *WNT11* on the facial phenotypes*.* As a reference, a face with average proportions is provided in Figure 2A.

rs17563 in *BMP4* was associated with lip proportion. Individuals with the homozygote GG genotype had a higher Ch-Ch:Ls-Li ratio than the heterozygote AG genotype (*p* = 0.034) (Table 3). Patients carrying the AG genotype had thicker lips (Figure 2B).

rs1533767 in *WNT11* was associated with the mid to lower face height ratio, in which patients carrying the GG genotype had larger lower face height compared to the AG genotype: G-Sn:Sn-Gn (*p* = 0.028) and N-Gn:Sn-Gn (*p* = 0.035). The results are shown in Table 3. Patients with the GG genotype are represented in Figure 2C.

rs1533767 in *WNT11* was associated with the ratio of the lower face height and the total face height (Sn-Gn:Tr-Gn). Table 3 shows that patients carrying the GG genotype had a larger lower face compared to the AG genotype (*p* = 0.039). Figure 2D represents patients carrying the GG genotype.

## 4 Discussion

Only recently have scientists started identifying the specific genes that impact the normal range of human facial morphology. As in the current study, previous studies applied a candidate gene approach to investigate whether genetic variants in genes were related to craniofacial development and therefore involved in one or more metric and non-metric facial traits (26). Gene selection is typically based on prior knowledge of their biological relevance, such as their expression during craniofacial development or being causal for a syndrome with craniofacial alterations resulting from single-gene mutations. SNPs in genes involved with syndromes were previously associated with a variety of facial measurements, for example, genes such as *FGFR1* (e.g., Pfeiffer syndrome and Kallmann syndrome) (3), *IRF6* (e.g., Van der Woude syndrome) (5), and *RUNX2* (e.g., cleidocranial dysplasia syndrome) (4). Some genome-wide association studies (GWASs) have also been performed and suggested some *loci* and SNPs (2, 6, 7). Other studies selected the candidate genes based on their role in oral cleft formation (1, 8). This is a similar approach used in our study, which selected SNPs in genes previously associated with oral clefts.

One of the most remarkable pieces of evidence that supports the association between oral cleft-related genes and normal face variability is the fact that the faces of unaffected parents of subjects with oral clefts display meaningful shape differences compared with the general population (8, 26). Indencleef et al. (8) conducted an interesting study design in which they studied unaffected parents of patients with non-syndromic oral clefts and control subjects. They found that some *loci* associated with oral cleft risk are also involved in some facial phenotypes. In our study, none of the included patients reported first-degree relatives with oral clefts.

The SNPs rs235768 (*BMP2*) and rs17563 (*BMP4*) were suggested as risk factors for oral cleft in the Iranian population (11). In our study, rs235768 in *BMP2* was not associated with the studied facial proportions, however, rs17563 in *BMP4* was associated with the Ch-Ch:Ls-Li ratio (lip proportion). A meta-analysis that pooled the results for this SNP supported that rs17563 may be associated with the risk for oral cleft development (13). Interestingly, this SNP was involved in lip proportion, as individuals carrying the GG genotype have a thinner lip, while patients carrying the AG genotype have thicker lips, supporting that this SNP may have an important role in perioral development.

A complex signaling network involving *BMPs* and *WNTs* in the facial ectoderm and neural crest mesenchyme is involved in the morphogenesis of the upper jaw (20). *WNT* signaling interacts with *BMP* to regulate the patterning and growth of the craniofacial skeleton (27). We observed that the SNP rs1533767 in *WNT11* was associated with the following facial proportions: the mid and lower face height ratio (G-Sn:Sn-Gn and N-Gn:Sn-Gn) and the ratio of the lower face height and the total face height of all three facial thirds (Sn-Gn:Tr-Gn). Previously, this SNP was borderline associated with a brachyfacial profile diagnosed using lateral cephalometric analysis to investigate craniofacial patterns (28). Chiquet et al. (9) also found that the SNP rs1533767 was associated with isolated oral cleft.

The current study has some obvious limitations that should be discussed here. The first to be highlighted is the fact that two-dimensional photographs were used in the phenotype evaluation. Although this method is still used in different studies, a facial scan followed by a three-dimensional analysis brings more reliable data. The fact that only a few candidate genes and SNPs were selected is also a limitation. Several SNPs in these genes and in other oral cleft-related genes may play an important role in facial morphology. It is important to mention that candidate gene studies, such as our study, frequently only examine a small number of genes/SNPs that are hypothesized to be relevant based on previous research, as studying a large number of genes requires significant time, funding, and resources. In addition, analyzing several genes/SNPs increases the risk of false-positive findings. A smaller, well-defined set of genes allows for more robust statistical analysis and validation of results. Focusing on fewer SNPs simplifies data analysis and makes it easier to draw meaningful biological conclusions, especially in small samples.

Another important aspect to be mentioned is the sample size. Although a sample size calculation was performed based on the frequency of the common genotype (wild homozygotic), the contribution of genotypes with a low frequency in the phenotypic variation could not be tested. The fact that the study was performed using two-dimensional photographs instead of a three-dimensional photograph should also be mentioned as a limitation of the current study. When studying facial morphology, two-dimensional images have some limitations compared to three-dimensional imaging, including a lack of depth perception, as two-dimensional images capture only height and width, making it difficult to analyze facial depth and curvature. Two-dimensional images also overlap structures and can obscure some important details. In contrast, three-dimensional imaging allows for direct, precise volumetric and angular measurements. Therefore, future studies with a larger cohort sample should be performed to confirm the findings of the present study in a Brazilian population and other populations.

Briefly, the discovery of genes involved in facial morphology has significant clinical relevance, particularly in orthodontics and personalized dental care. Future genetic screening can help predict facial morphology, guiding treatment plans for specific phenotypes before they fully develop. In the long term, discoveries in craniofacial genetics could lead to gene-based therapies.

## 5 Conclusion

Our study supported the hypothesis that oral cleft-related genes are involved in the normal range of variations in the human face. The SNPs rs17563 in *BMP4* and rs1533767 in *WNT11* were associated with facial proportion variations. Understanding the genetic basis underlying the normal-range variation of the human face has important implications in clinical studies, developmental biology, counseling, and forensic science. It is important to emphasize that the present genetic study was conducted with a relatively small sample size, which may limit the generalizability and statistical power of the findings. While the results provide valuable insights regarding the genetic background of the normal range of variation of the human face, future studies with larger cohorts are necessary to validate these findings, improve the accuracy of genetic associations, and strengthen the reliability of conclusions drawn from this research. Expanding sample sizes will also help to identify subtle genetic effects and enhance our understanding of the complex interplay between genetics and human face variability.

## Data Availability

The datasets presented in this study can be found in online repositories. The names of the repository/repositories and accession number(s) can be found in the article/Supplementary Material.

## References

[1] Boehringer S van der Lijn F Liu F Günther M Sinigerova S Nowak S Genetic determination of human facial morphology: links between cleft-lips and normal variation. Eur J Hum Genet. (2011) 19(11):1192–7. 10.1038/ejhg.2011.110 21694738 PMC3198142

[2] Paternoster L Zhurov AI Toma AM Kemp JP Pourcain BS Timpson NJ Genome-wide association study of three-dimensional facial morphology identifies a variant in PAX3 associated with nasion position. Am J Hum Genet. (2012) 90(3):478–85. 10.1016/j.ajhg.2011.12.021 22341974 PMC3309180

[3] Gómez-Valdés JA Hünemeier T Contini V Acuña-Alonzo V Macin G Ballesteros-Romero M Fibroblast growth factor receptor 1 (FGFR1) variants and craniofacial variation in Amerindians and related populations. Am J Hum Biol. (2013) 25(1):12–9. 10.1002/ajhb.22331 23070782

[4] Adhikari K Fontanil T Cal S Mendoza-Revilla J Fuentes-Guajardo M Chacón-Duque JC A genome-wide association scan in admixed Latin Americans identifies loci influencing facial and scalp hair features. Nat Commun. (2016) 1(7):10815. 10.1038/ncomms10815 PMC4773514 26926045

[5] Weinberg SM Naidoo SD Bardi KM Brandon CA Neiswanger K Resick JM Face shape of unaffected parents with cleft affected offspring: combining three-dimensional surface imaging and geometric morphometrics. Orthod Craniofac Res. (2009) 12(4):271–81. 10.1111/j.1601-6343.2009.01462.x 19840279 PMC2765674

[6] White JD Indencleef K Naqvi S Eller RJ Hoskens H Roosenboom J Insights into the genetic architecture of the human face. Nat Genet. (2021) 53(1):45–53. 10.1038/s41588-020-00741-7 33288918 PMC7796995

[7] Xiong Z Gao X Chen Y Feng Z Pan S Lu H Combining genome-wide association studies highlight novel loci involved in human facial variation. Nat Commun. (2022) 13(1):7832. 10.1038/s41467-022-35328-9 36539420 PMC9767941

[8] Indencleef K Hoskens H Lee MK White JD Liu C Eller RJ The intersection of the genetic architectures of orofacial clefts and normal facial variation. Front Genet. (2021) 12:626403. 10.3389/fgene.2021.626403 33692830 PMC7937973

[9] Chiquet BT Blanton SH Burt A Ma D Stal S Mulliken JB Variation in WNT genes is associated with non-syndromic cleft lip with or without cleft palate. Hum Mol Genet. (2008) 17(14):2212–8. 10.1093/hmg/ddn121 18413325 PMC2852032

[10] Antunes LS Küchler EC Tannure PN Costa MC Gouvêa CVD Olej B BMP4 Polymorphism is associated with nonsyndromic oral cleft in a Brazilian population. Cleft Palate Craniofac J. (2013) 50(6):633–8. 10.1597/12-048 23237431

[11] Saket M Saliminejad K Kamali K Moghadam FA Anvar NE Khorshid HRK. BMP2 and BMP4 variations and risk of non-syndromic cleft lip and palate. Arch Oral Biol. (2016) 72:134–7. 10.1016/j.archoralbio.2016.08.019 27591802

[12] Kiranahayu R Suhartono AW Sulistyani LD Latief BS Aue EI. Association of *rs235768* A > T polymorphism of the bone morphogenetic protein 2 gene on non-syndromic orofacial cleft in an Indonesian population. Padj J Dent. (2020) 32(2):136–41. 10.24198/pjd.vol32no2.23917

[13] Bahrami R Dastgheib SA Niktabar SM Amooee A Lookzadeh MH Mirjalili SR Association of BMP4 *rs17563* polymorphism with nonsyndromic cleft lip with or without cleft palate risk: literature review and comprehensive meta-analysis. Fetal Pediatr Pathol. (2012) 40(4):305–19. 10.1080/15513815.2019.1707916 31909686

[14] Oliveira LQR de Souza Nicolau HC Barbosa Martelli DR Martelli-Júnior H Scariot R Ayroza Rangel ALC Ethnic differences in the Brazilian population influence the impact of BMP4 genetic variants on susceptibility of nonsyndromic orofacial clefts. Cleft Palate Craniofac J. (2024) 61(10):1701–12. 10.1177/10556656231180086 37272066

[15] Minoux M Rijli FM. Molecular mechanisms of cranial neural crest cell migration and patterning in craniofacial development. Development. (2010) 137(16):2605–21. 10.1242/dev.040048 20663816

[16] Ritzman TB Banovich N Buss KP Guida J Rubel MA Pinney J Facing the facts: the Runx2 gene is associated with variation in facial morphology in primates. J Hum Evol. (2017) 111:139–51. 10.1016/j.jhevol.2017.06.014 28874267

[17] Graf D Malik Z Hayano S Mishina Y. Common mechanisms in development and disease: BMP signaling in craniofacial development. Cytokine Growth Factor Rev. (2016) 27:129–39. 10.1016/j.cytogfr.2015.11.004 26747371 PMC4753105

[18] Liu W Sun X Braut A Mishina Y Behringer RR Mina M Distinct functions for bmp signaling in lip and palate fusion in mice. Development. (2005) 132(6):1453–61. 10.1242/dev.01676 15716346

[19] Bennett JH Hunt P Thorogood P. Bone morphogenetic protein−2 and −4 expression during murine orofacial development. Arch Oral Biol. (1995) 40(9):847–54. 10.1016/0003-9969(95)00047-s 8651889

[20] Marchini M Hu D Vercio LL Young NM Forkert ND Hallgrímsson B *Wnt* signaling drives correlated changes in facial morphology and brain shape. Front Cell Dev Biol. (2021) 9:644099. 10.3389/fcell.2021.644099 33855022 PMC8039397

[21] Brugmann SA Powder KE Young NM Goodnough LH Hahn SM James AW Comparative gene expression analysis of avian embryonic facial structures reveals new candidates for human craniofacial disorders. Hum Mol Genet. (2010) 19(5):920–30. 10.1093/hmg/ddp559 20015954 PMC2816616

[22] Lee JM Kim JY Cho KW Lee MJ Cho SW Kwak S Wnt11/Fgfr1b cross-talk modulates the fate of cells in palate development. Dev Biol. (2008) 314(2):341–50. 10.1016/j.ydbio.2007.11.033 18191119

[23] Aberg T Cavender A Gaikwad JS Bronckers ALJJ Wang X Waltimo-Sirén J Phenotypic changes indentition of Runx2 homozygote-null mutant mice. J Histochem Cytochem. (2004) 52(1):131–9. 10.1177/002215540405200113 14688224

[24] Wu T Fallin MD Shi M Ruczinski I Liang KY Hetmanski JB Evidence of gene-environment interaction for the RUNX2 gene and environmental tobacco smoke in controlling the risk of cleft lip with/without cleft palate. Birth Defects Res A Clin Mol Teratol. (2012) 94(2):76–83. 10.1002/bdra.22885 22241686 PMC3608124

[25] Küchler EC Tannure PN Falagan-Lotsch P Lopes TS Granjeiro JM Amorim LMF. Buccal cells DNA extraction to obtain high quality human genomic DNA suitable for polymorphism genotyping by PCR-RFLP and real-time PCR. J Appl Oral Sci. (2012) 20(4):467–71. 10.1590/s1678-77572012000400013 23032210 PMC3881822

[26] Peng S Tan J Hu S Zhou H Guo J Jin L Detecting genetic association of common human facial morphological variation using high density 3D image registration. PLoS Comput Biol. (2013) 9(12):e1003375. 10.1371/journal.pcbi.1003375 24339768 PMC3854494

[27] Alexander C Piloto S Pabic PL Schilling TF. Wnt signaling interacts with bmp and edn1 to regulate dorsal-ventral patterning and growth of the craniofacial skeleton. PLoS Genet. (2014) 10(7):e1004479. 10.1371/journal.pgen.1004479 25058015 PMC4109847

[28] Küchler EC Reis CLB Carelli J Scariot R Nelson-Filho P Coletta RD Potential interactions among single nucleotide polymorphisms in bone- and cartilage-related genes in skeletal malocclusions. Orthod Craniofac Res. (2021) 24(2):277–87. 10.1111/ocr.12433 33068497

